# Enhanced contact performance of high-brightness micro-LEDs via ITO/Al anode stack and annealing process

**DOI:** 10.1038/s41598-024-63075-y

**Published:** 2024-05-27

**Authors:** Zeyang Meng, Chaoyu Lu, Guanghua Wang, Sibo Gao, Feng Deng, Jie Zhang, Shuxiong Gao, Wenyun Yang

**Affiliations:** 1 Yunnan Olightek Opto-electronic Technology Co., Ltd., Kunming, 650223 China; 2 School of Materials and Energy, Yunnan University, Kunming, 650091 China; 3 Kunming Institute of Physics, Kunming, 650223 China

**Keywords:** Micro-LEDs, Anode contact, Electrode structure design, Materials science, Materials for devices

## Abstract

Micro-light-emitting diodes (Micro-LEDs) are a new type of display device based on the third-generation semiconductor gallium nitride (GaN) material which stands out for its high luminous efficiency, elevated brightness, short response times, and high reliability. The contact between anode layers and P-GaN is one of the keys to improving the performance of the devices. This study investigates the impact of electrode structure design and optimized annealing conditions on the anode contact performance of devices. The Micro-LED device with the size of 9.1 μm whose electrode structure is ITO/Ti/Al/Ni/Cr/Pt/Au (100/50/350/100/500/500/5000 Å) exhibits a significant improvement in contact performance after annealing under the Ar gas atmosphere at 500 °C for 5 min. The optimized device exhibited a current of 10.9 mA and a brightness of 298,628 cd/m^2^ under 5 V. The EQE peak value of Device A is 10.06% at 400 mA.

## Introduction

**Figure 1:**
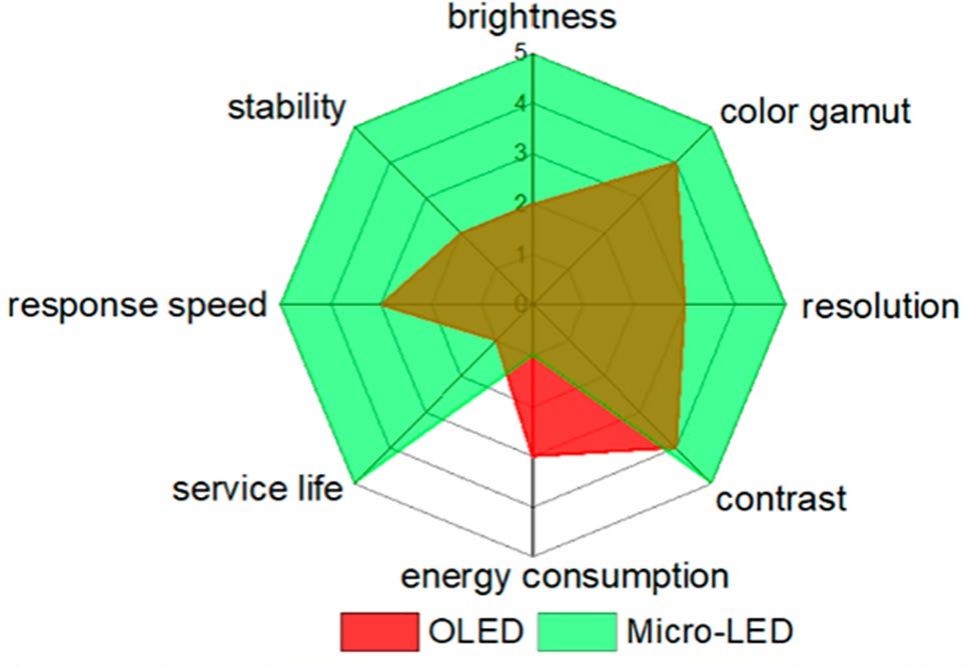
Radar chart of OLED vs. Micro-LED in several aspects^25^.

Displays composed of micro-light-emitting diodes (Micro-LEDs) are regarded as promising next-generation self-luminous screens and have advantages such as high brightness, high color purity, long lifetime^1–3^. In contrast to the Organic Light Emitting Diode (OLED) display depicted in Fig. 1, Micro-LED devices exhibit both elevated luminance and responsiveness. Therefore, Micro-LED can be widely applied in mobile phones^4,5^, high-precision optoelectronic detectors^6–11^, VR/AR devices^12–16^, high-speed optical communication^17–20^, medical research^21–24^ and so on. However, many technical difficulties and challenges on the road to commercialization need to be overcome. The fabrication of Micro-LED devices is the foundation of commercialization.

The fabrication of Micro-LED devices aims to miniaturize traditional large-sized LEDs to below 100 µm and integrates them in an array format on circuits. The research on the fabrication technology of Micro-LED devices currently in remains its infancy. Some basic scientific and technological problems in micro-LED devices remain to be resolved, such as the reduction of device defect density, optimization of device heat dissipation, repair of sidewall damage, and improvement of anode-electrode contact^26^. All these problems influence the optoelectronic performance of the Micro-LED devices. How to improve the anode contact is one of the key focuses to improve the Micro-LED performance.

The metal anode structure includes a current spreading layer (CSL), a reflective layer, a blocking layer, and a bonding layer. The CSL enhances the contact performance between the metal and P-GaN. The reflective layer contributes to the improvement of Micro-LED light extraction efficiency^27^, commonly used reflective layer metals with high visible light reflectance include Ag and Al. The blocking layer is employed to prevent the diffusion of metals from the bonding layer into the reflective layer and the active layer of the device. On the other hand, the bonding layer is utilized to establish a stable electrical connection between the Micro-LED chip and the driving circuit. In recent years, many research works have already made efforts to optimize the anode contact.

In 2021, Liu et al. employed a Ni/Au (5 nm/5 nm) layer stack as the CSL and achieved Ohmic contact between P-GaN and CSL through rapid thermal annealing (RTA)^28^. Their experiment is designed to study the impact of annealing processes on the electrical performance of Micro-LED devices at different temperatures (303–573 K). On the other hand, Li et al. deposited Ni/Au (4 nm/4 nm) as the CSL layer. They achieved Ohmic contact between P-GaN and CSL by the annealing process^29^. They also investigated the influence of temperature on the Ohmic contact between the anode electrode and P-GaN by employing temperature-dependent transmission line model (TLM) measurements. Zhang et al. added ITO as CSL provided excellent device performance. Over 10% external quantum efficiency (EQE) and wall-plug efficiency (WPE), and ultra-high brightness (> 10 M nits) green micro-LEDs are realized in their experiment^30^.

**Figure 2:**
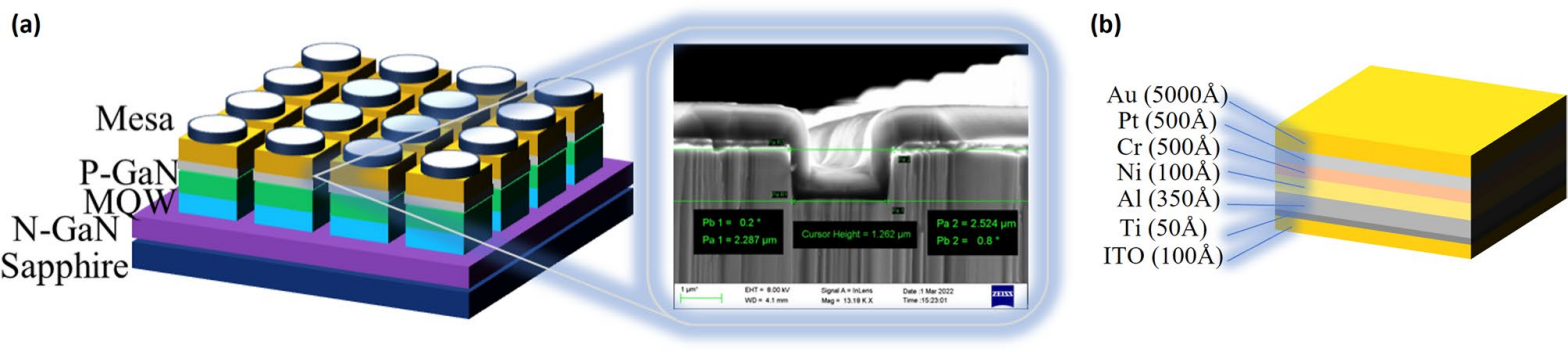
(**a**) The structure and the SEM characterization of the Micro-LED chip. (**b**) The anode structures of the device.

To further enhance the anode contact of the device and improve device optoelectronic performance, in this work, the high work function of ITO layer and high reflectivity of Al layer were added to the anode structure as a part of the CSL and the reflective layer which will achieve good ohmic contact with P-GaN. The Ti (5 nm) in the middle of ITO and Al bind them more firmly. The Ni deposited on the Al will protect the ITO and Al from being oxidized. The Cr/Pt with the thickness of 50/50 nm alloys were deposited as the blocking layer to prevent the diffusion of metal atoms into the active region of the LED device during the high-temperature annealing process^31^. The Au with the thickness of 500 nm improves the conductivity and corrosion resistance of the electrode. The structure of the device is showed in Fig. 2a and the anode structures of the single pixel is showed in Fig. 2b. This paper deeply explored the advantages of ITO and Al as anode materials. We also studied the impact of the annealing process on the performance of devices. Through this study, we reduced the turn-on voltage of Micro-LED devices, improved the contact between the semiconductor and metal, and optimized the design strategy for device electrode structures. This provides theoretical support and design ideas for the industrial production of Micro-LED devices.

## Methods

**Figure 3:**
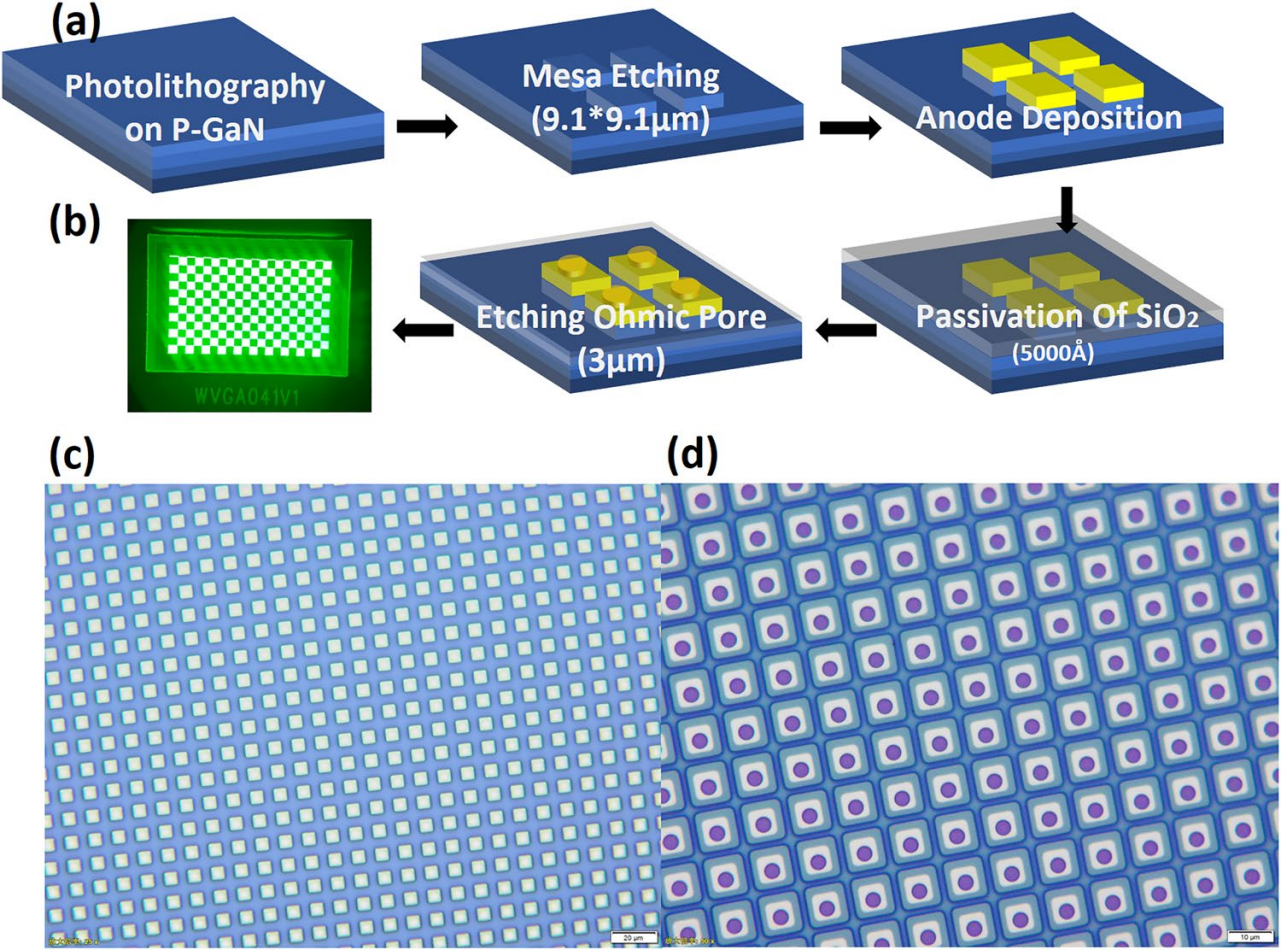
The fabrication process and optical micrograph of the Micro-LED. (**a**) Schematic diagram of Micro-LED fabrication process. (**b**) The Micro-LED device. (**c**) Optical micrograph of the chip. (**d**) Optical micrograph of the mesa with SiO_2_.

The schematic diagram of the micro-LED fabrication processes is shown in Fig. 3a. The micro-LED devices are fabricated on a standard GaN green LED epi-wafer. The micro-LED pixels (9.1 μm) are fabricated through the photolithography process, which starts with mesa etching by IBE for 2 min. The anode is then deposited for the P-GaN ohmic contact. Next, the devices are passivated by a 500 nm SiO_2_ layer deposited by plasma-enhanced chemical vapor deposition, which is followed by contact window opening by IBE. Finally, thick metal stacks based on the Cr/Pt/Au layers are used for the N-GaN ohmic contact. We designed three anode structures called A, B, and C to analyze the effect of anode structure on the device. Then, two other samples were designed to study the effect of annealing temperature on ohmic contact. We annealed the chips to achieve ohmic contact under the Ar gas atmosphere at 500 °C (400 °C) for 5 min. By using the dicing machine, the chip and IC were cut into complete Dies. Subsequently, the chip was connected to the IC circuit by using the flip-chip bonder. (Supplementary methods 1.1–1.6)The lighting effect of device A is shown in Fig. 3b. The anode structures and annealing temperatures of all samples involved in this paper are shown in Table 1.

**Table 1:** The micro-LED devices used for experiments.

Micro-LED sample label	Electrode structure	Annealing temperature (°C)
A	ITO/Ti/Al/Ni/Cr/Pt/Au (100/50/350/100/500/500/5000 Å)	500
B	Ti/Al/Ni/Cr/Pt/Au (50/350/100/500/500/5000 Å)	500
C	ITO/Ti/Ni/Cr/Pt/Au (100/50/100/500/500/5000 Å)	500
D	ITO/Ti/Al/Ni/Cr/Pt/Au (100/50/350/100/500/500/5000 Å)	400
E	ITO/Ti/Al/Ni/Cr/Pt/Au (100/50/350/100/500/500/5000 Å)	Unannealed

## Results and discussion

**Figure 4:**
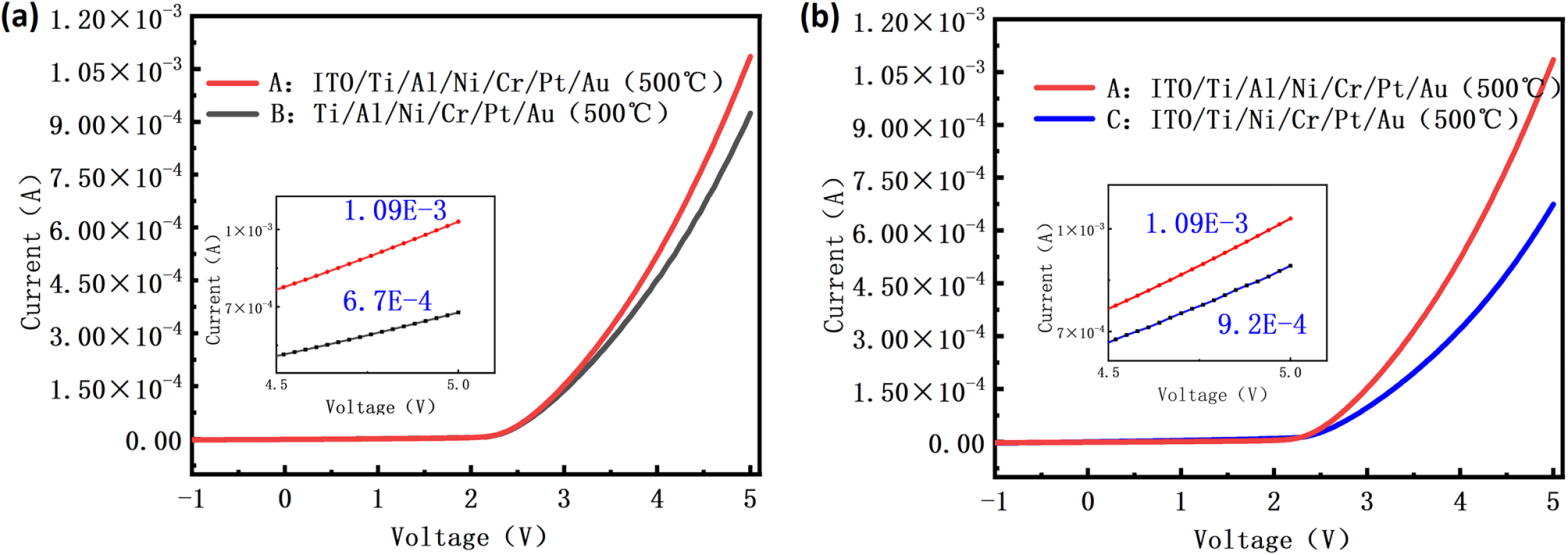
The current–voltage characteristics of the devices. (**a**) The current–voltage curves of device A and device B. (**b**) The current–voltage curves of sample A and sample C.

As can be seen from the optical microscope in Fig. 2c,d, the photolithography process and etching process achieved pixel isolation. The size of the pixel is 9.1 μm, and the pixel pitch is about 2 μm. As mentioned above, the ITO layer possesses high conductivity, optical transparency, and a wide voltage response range^32^, making it an ideal CSL for Micro-LED. The current against the voltage is compared in Fig. 4a. The turn-on voltage for Device A is 2.51 V, the current of Device A is 10.9 mA at 5 V and the current of Device B is 9.24 mA (Supplementary Fig. 1s). The current increases by 18% from Device A to Device B at 5 V because of the improved contact performance between the ITO layer and P-GaN. This can be attributed to the fact that the ITO layer leads to an Ohmic contact by forming a p–n junction at the interface of ITO/P-GaN and induces the carrier transport by tunneling from the ITO to P-GaN. The contact interface is influenced by the work function of the ITO, redistributing the charge states at the interface. These charge states impact the built-in electric field, assisting in regulating the carrier transport in the heterojunction. During the contact between the anode and the P-GaN, free electrons in ITO will enter P-GaN, forming electron–hole pairs. These electron–hole pairs reduce the barrier height for carrier transport, making it easier for carriers to transfer in the device^33^. The energy band bending that happened at the ITO and P-GaN contact interface also reduces the injection barrier for holes in P-GaN, making it easier for holes to inject into the device^34^. The addition of ITO layer enhances the injection efficiency of the holes, increases the electrical performance of the device.

**Figure 5:**
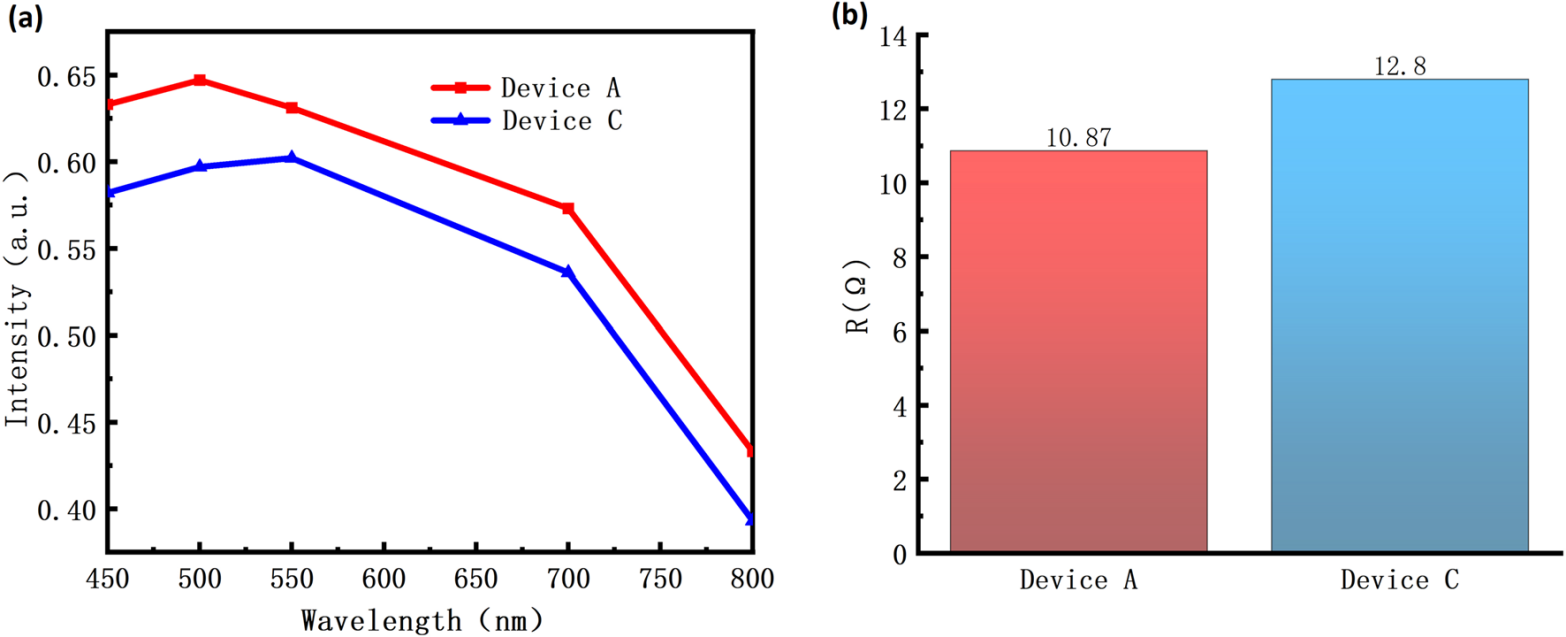
(**a**) The reflectivity test results for the contact layer portion of Device A and Device C. (**b**) The square resistance test results of Device A and Device C.

The current–voltage plot between Device A and Device C is shown in Fig. 4b to investigate the impact of the reflective layer with Al on device performance. The turn-on voltage of the Device C is 2.54 V and the current of Device C is 6.74 mA at 5 V which is 61.7% lower than Device A. As the square resistance measurement shows in Fig. 5b, the square resistance value of the anode structure alloys for Device A is 10.87 Ω, which is less than the square resistance values of the anode structure alloy for Device C. The reflectance testing results in Fig. 5a indicate that the reflectance of the Device A is higher than the Device C. One aspect contributing to the performance improvement is the high conductivity of Al, which can reduce the device's resistance, thereby enhancing the light efficiency of the device^35^. Another aspect is that, during the annealing process, Al atoms diffuse from the reflective layer into the ITO layer. This portion of Al replaces In or Sn in ITO, forming an Al-doped ITO film^36^. The forming of the Al-doped ITO film results in lower contact resistance and more uniform current distribution in the composite electrode structure after annealing, thereby improving the device's electrical performance.

The specific contact resistivity of three distinct electrodes was measured by the Transmission Line Model (TLM) technique, with the resultant data presented in Table 2. The specific contact resistivity of Device A (ITO/Ti/Al/Ni/Cr/Pt/Au), Device B (Ti/Al/Ni/Cr/Pt/Au), and Device C (ITO/Ti/Ni/Cr/Pt/Au) were 7.803 × 10^−4^ Ω cm^2^, 4.682 × 10^−3^ Ω cm^2^, and 5.689 × 10^−1^ Ω cm^2^, respectively. The results in Table 2 are consistent with the results in Fig. 4, where Device A has the smallest specific contact resistivity and therefore the best electrical performance. By comparing the specific contact resistivity between Device A and Device B, Device A and Device C, it is concluded that the contact performance of the Micro-LED devices can be improved by adding ITO and Al to the Micro-LED anode electrode (Supplementary Information).

**Table 2:** The result of the contact resistance between the electrode and P-GaN.

Micro-LED sample label	Electrode structure	Specific contact resistivity with P-GaN
A	ITO/Ti/Al/Ni/Cr/Pt/Au (100/50/350/100/500/500/5000 Å)	7.803 × 10^−4^ Ω cm^2^
B	Ti/Al/Ni/Cr/Pt/Au (50/350/100/500/500/5000 Å)	4.682 × 10^−3^ Ω cm^2^
C	ITO/Ti/Ni/Cr/Pt/Au (100/50/100/500/500/5000 Å)	5.689 × 10^−1^ Ω cm^2^

**Figure 6:**
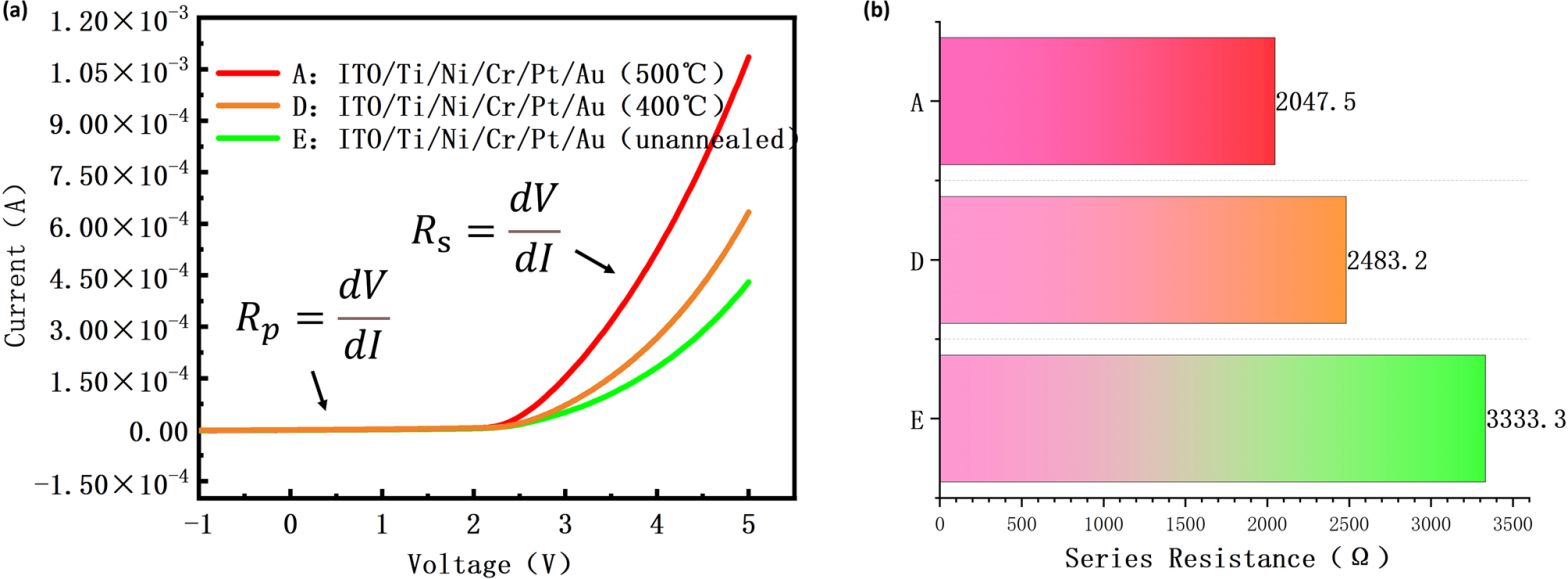
The electrical properties of the devices with different annealing temperatures. (**a**) The current–voltage curves of the devices in the calculation. (**b**) The diagram of the calculated series resistance of samples A, D, and E.

As described above, the annealing process has an impact on the electrical performance of the device. We compared three different devices with different annealing temperatures (500 °C, 400 °C) in their performance. As shown in Fig. 6a, among devices annealed at different temperatures with the same anode structure, Device A exhibits the highest current under the voltage of 5 V (Supplementary Fig. 2s). The great electrical performance of Device A is attributed to the effective repair of the sidewall damage caused during the etching process^37^. During the annealing process, defects in the metal lattice are repaired, and the impurity atoms are activated by the high temperature to fill the damaged sites. That is the reason why the diffusion of impurities is minimized during the annealing process. The defects of the ITO film also reduced during the recrystallization process when the device was in the annealing process^38^.

Subsequently, we calculated the series resistance and ideality factor values (n) of devices A, D, and E to further study their electrical characteristics. For the P–N junction of a Micro-LED chip, the ideal current–voltage relationship can be described by the Shockley Eq. (1). Here, n is the LED ideality factor; e is the elementary charge (e = 1.6 × 10^−19^ C); k is the Boltzmann constant (k = 1.380649 × 10^−23^ J/K); T is the thermodynamic temperature.

1 $$I = I_{s} e^{ev/(nkT)}$$

In practical LED chips, considering the influence of series resistance and parallel resistance on the current–voltage characteristics of LEDs, the Shockley equation has been modified as follows (2):

2 $$I - \frac{{(V - IR_{s} )}}{{R_{p} }} = I_{s} e^{{e(V - IR_{s} )/(nkT)}}$$

3 $$R_{p} = \frac{dV}{{dI}}$$

4 $$R_{s} = \frac{dV}{{dI}}$$

5 $$n = \frac{e}{kT}\left( {\frac{\partial lnI}{{\partial V}}} \right)^{ - 1}$$

Here, R_s_ is the series resistance, and R_p_ is the parallel resistance. When the driving voltage is less than the threshold voltage, the parallel resistance R_p_ can be calculated using the following Eq. (3)^39^. On the other hand, when the driving voltage is higher than the turn-on voltage, the series resistance R_s_ can be calculated using the following Eq. (4).

The calculation results indicate that the ideality factors (n) of devices A, D and E are all greater than 2. The results argue that the transport mechanism of the carriers in the devices is the tunneling effect of defect replication^40^. The carriers can tunnel through barriers via the tunneling effect, entering the other side of the semiconductor material. This process typically occurs near defects in the semiconductor material, as these locations have lower barriers and are more conducive to the electrical performance of the device^41,42^.

As shown in Fig. 6a, the current value of all devices increases as the voltage increases, with Device A having the largest slope because the carrier concentration increases. With the higher annealing temperature, the carrier concentration increases due to the release of electrons from ITO^43^. The mobility of the ITO improved when the films were annealed at 400 °C and 500 °C, due to the proper reordering of grains, decrease in strain and dislocation density^44^.

As shown in Fig. 6b, we calculated the total series resistance of Device A, Device D and Device E between the voltage from 4.0 to 4.3 V by using the Eq. (4). The series resistance of Device A is reduced by 435.7 Ω compared to Device D; The series resistance of Device A is reduced by 1285.8 Ω compared to Device E. The result argues that the series resistance and the contact performance of the device are better when the device is annealed at 500 °C.

**Figure 7:**
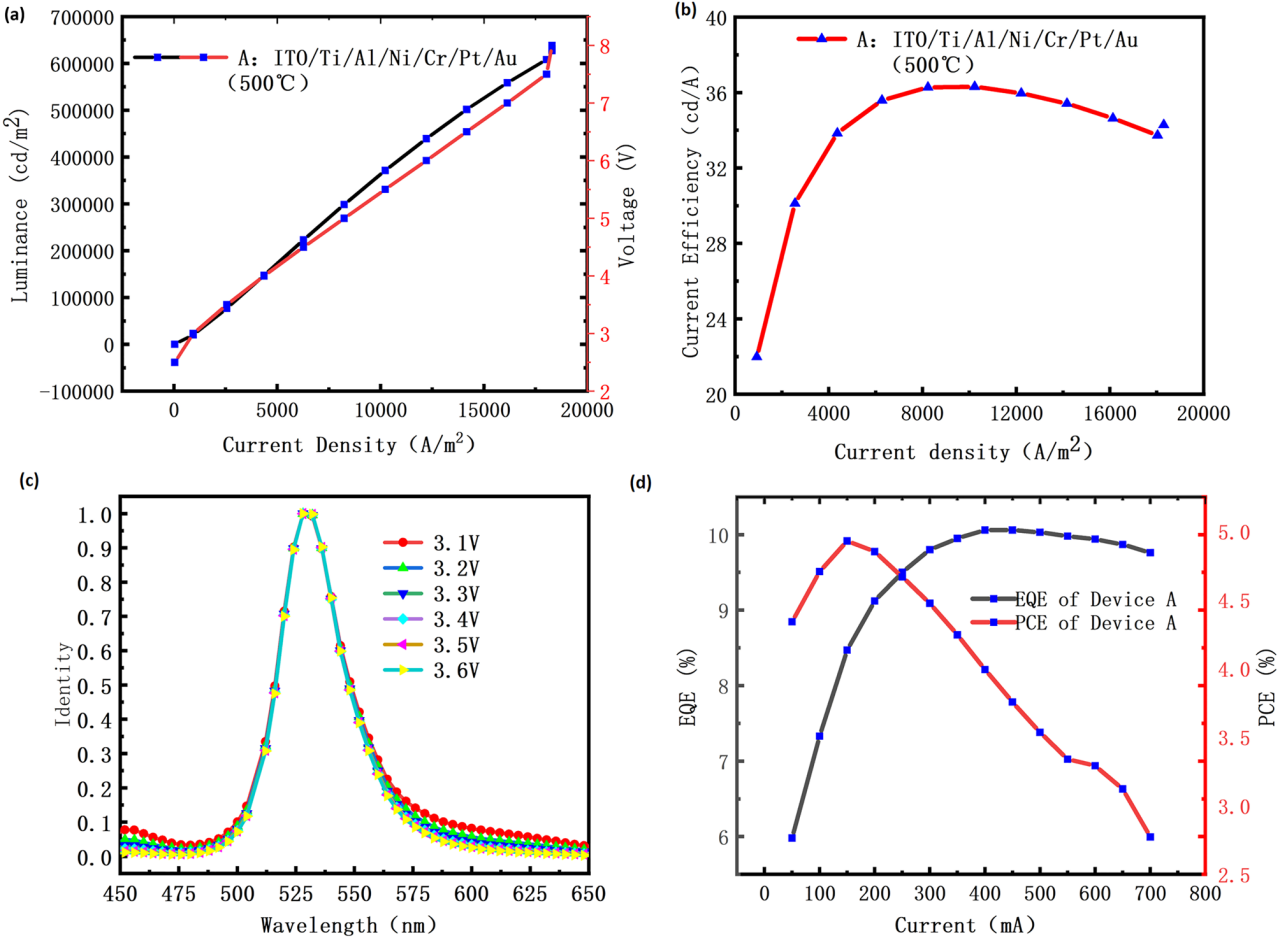
The optical properties of the Device A. (**a**) The light brightness-voltage graph of Device A. (**b**) The efficiency-current density graph of Device A. (**c**) The normalized emission wavelength-voltage graph of Device A. (**d**) The EQEs and PCEs of Device A.

The optical properties of Device A were characterized and analyzed by using the color photometer. We measured the pixel brightness of Device A over a certain area (12,494 pixels) to obtain data on the optical performance. As shown in Fig. 7a, the brightness of Device A is 298,628 cd/m^2^ at 5 V and gets to 627,214 cd/m^2^ at 8 V. In Fig. 7b, the peak current efficiency of Device A is 36.31%. In Fig. 7c, it can be observed that the emission wavelength of Device A is 528 nm (Supplementary Fig. 3s). The device in question exhibits a minimal shift in the wavelength of its principal peak, which indicates high precision in its optical performance and low defective density. This stability suggests that the device maintains consistent performance over time, which is critical for applications requiring reliability and reproducibility. Furthermore, the low incidence of defects implies robust manufacturing quality and potentially longer lifespan due to reduced wear and tear or failure rates. These attributes make it suitable for use in environments where accuracy and dependability are paramount, such as in scientific research, medical diagnostics, or industrial process control. In Fig. 7d, The EQEs and PCEs were measured using the calibrated integrating sphere. It can be seen that the EQEs peak at 400 mA (10.06%) and then drop with the increasing current because of the Quantum efficiency droop. As shown in Fig. 7d, the PCEs peak at 150 mA (4.92%) and then drop with the increasing current too. The reason why the PCEs curve of Micro-LED rises first and then decreases is related to the size effect of Micro-LED. As the size of a micro-LED decreases, the ratio of its surface area to volume increases, resulting in more sidewall damage and defects.

## Conclusion

The Micro-LED device with the anode structure of ITO/Ti/Al/Ni/Cr/Pt/Au exhibited outstanding performance. The current of the device reaches 10.9 mA and the brightness of the device gets to 298,628 cd/m^2^ at 5 V. The ITO and Al layers effectively improve the contact performance between the anode electrode and P-GaN, thereby enhancing the performance of the Micro-LED devices. The high-temperature annealing process repairs damages produced during the etching process which will significantly enhance the reliability of Micro-LED devices. The contribution of this paper will improve the design of the anode structure and contribute to the commercialization of the Micro-LED devices.

### Supplementary Information

Supplementary Information.

## Data Availability

The data that support the plots within this article and other findings of this study are available from the corresponding author upon reasonable request.
