# Supplementary material for: Enhanced contact performance of high-brightness micro-LEDs via ITO/Al anode stack and annealing process
Source: Sci Rep. 2024 May 27;14:12050. doi: 10.1038/s41598-024-63075-y (PMC11130201; doi:10.1038/s41598-024-63075-y)
Supplement: Supplementary file 1 — Supplementary Information. [file 41598_2024_63075_MOESM1_ESM.pdf]

1.Supplementary Methods

1.1 Photolithography

The grown gallium nitride (GaN) wafers on sapphire substrates are placed in a cleaning basket and sequentially cleaned with acetone and isopropyl alcohol to remove surface impurities, which are then confirmed to be absent using an optical microscope. Subsequently, the clean GaN/sapphire substrates are spin-coated with photoresist, followed by the standard Photolithography process to transfer the pattern from the mask onto the GaN substrate material. The procedure concludes with exposure and development treatments.

1.2 Mesa Etching

The processed GaN substrates are introduced into an ion beam etching system(IBE) to perform the etching of pixel mesas. Following the etching process, the photoresist on the GaN wafers is removed with acetone, and the surfaces are thoroughly cleaned. The optical microscopy platform is utilized to confirm the absence of any residues post-cleaning. This completes the etching of the pixel array mesas on the GaN substrate material.

1.3 Anode Deposition

The etched chips with pixel mesas undergo a second round of photolithography to protect the etched pixels on their surfaces. After standard photolithographic processing, a sequential deposition of metallic electrodes is performed using magnetron sputtering, starting with a ITO/Ti/Al/Ni/Cr/Pt/Au(100/50/350/100/500/500/5000Å) stack.

1.4 Passivation Of SiO2

The chips are immersed in acetone to soften the photoresist, which is subsequently removed with an acetone gun. A layer of silicon dioxide is then deposited as a passivation layer.

1.5 Etching Ohmic Pore

Ohmic contacts on the chip are created through a process involving photolithography and IBE technology.

1.6 Connected to the IC

Lastly, a layer of ITO is grown on top as a conductive electrode after the ohmic contact holes have been opened. Subsequently, the chip was connected to the IC circuit by using the flip-chip bonder.

2.Supplementary figures

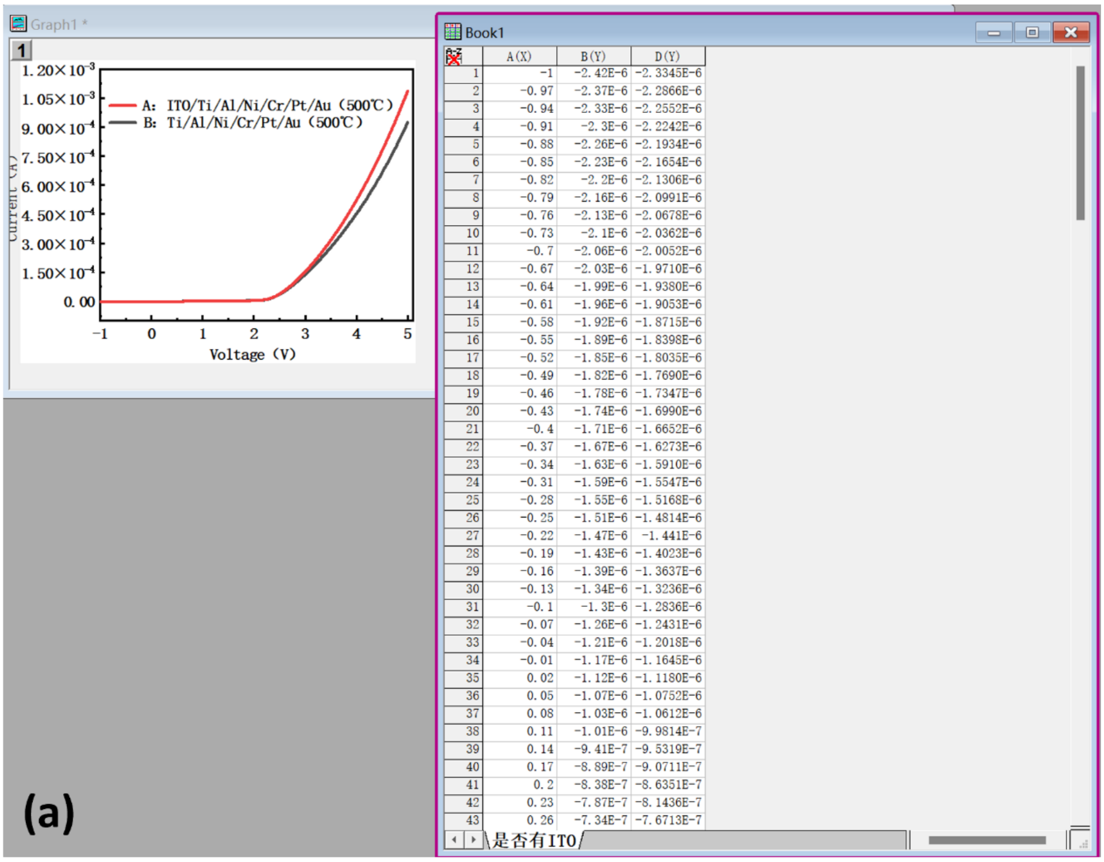

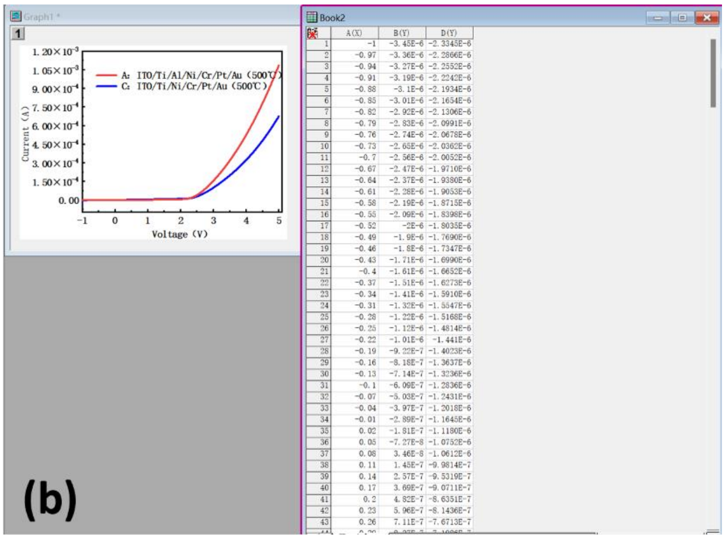

Figure 1s. The current-voltage characteristics of the devices. **(a)** The current-voltage curves of device A and device B. **(b)** The current-voltage curves of sample A and sample C.

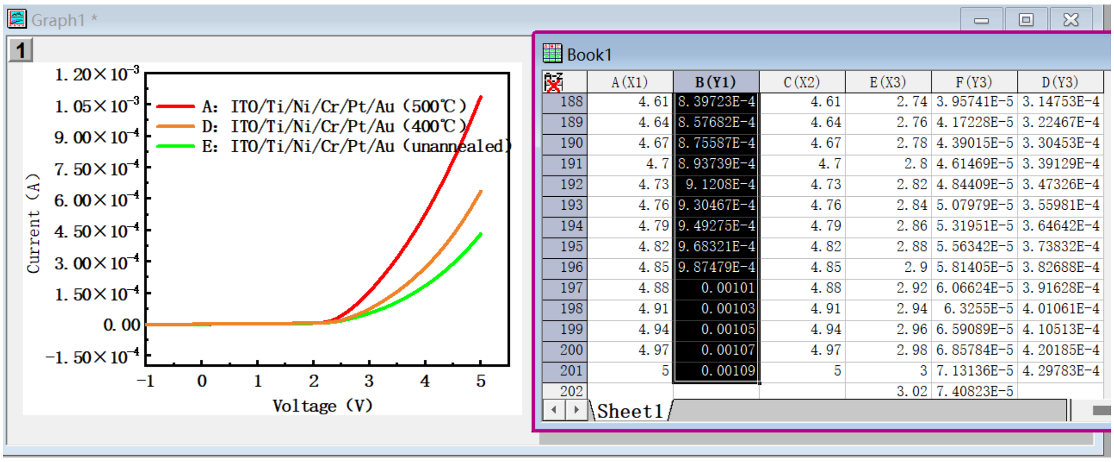

	A(X1)	B(Y1)	C(X2)	E(X3)	F(Y3)	D(Y3)
188	4.61	8.39723E-4	4.61	2.74	3.95741E-5	3.14753E-4
189	4.64	8.57682E-4	4.64	2.76	4.17228E-5	3.22467E-4
190	4.67	8.75587E-4	4.67	2.78	4.39015E-5	3.30453E-4
191	4.7	8.93739E-4	4.7	2.8	4.61469E-5	3.39129E-4
192	4.73	9.1208E-4	4.73	2.82	4.84409E-5	3.47326E-4
193	4.76	9.30467E-4	4.76	2.84	5.07979E-5	3.55981E-4
194	4.79	9.49275E-4	4.79	2.86	5.31951E-5	3.64642E-4
195	4.82	9.68321E-4	4.82	2.88	5.56342E-5	3.73832E-4
196	4.85	9.87479E-4	4.85	2.9	5.81405E-5	3.82688E-4
197	4.88	0.00101	4.88	2.92	6.06624E-5	3.91628E-4
198	4.91	0.00103	4.91	2.94	6.3255E-5	4.01061E-4
199	4.94	0.00105	4.94	2.96	6.59089E-5	4.10513E-4
200	4.97	0.00107	4.97	2.98	6.85784E-5	4.20185E-4
201	5	0.00109	5	3	7.13136E-5	4.29783E-4
202				3.02	7.40823E-5	

Figure 2s. The electrical properties of the devices with different annealing temperatures.

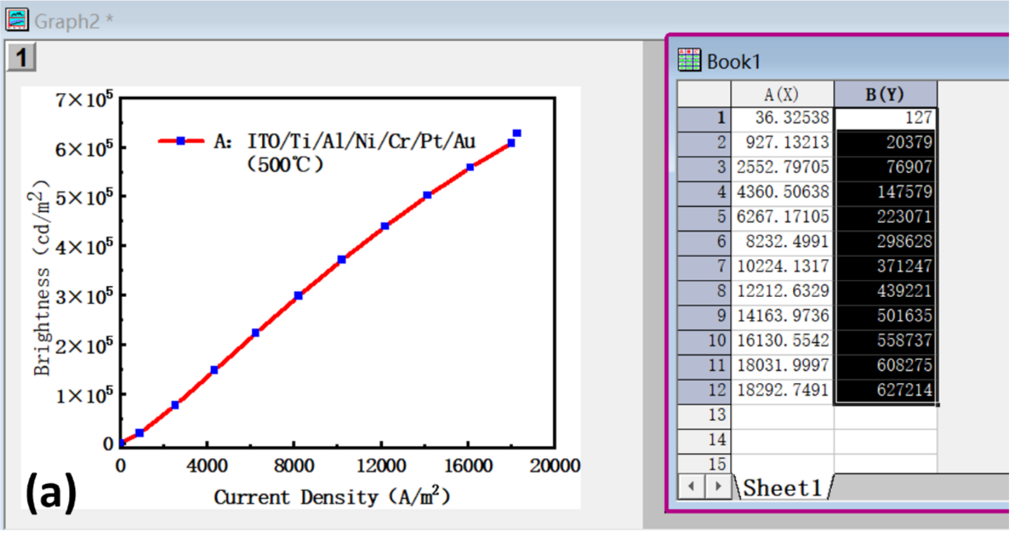

	A(X)	B(Y)
1	36.32538	127
2	927.13213	20379
3	2552.79705	76907
4	4360.50638	147579
5	6267.17105	223071
6	8232.4991	298628
7	10224.1317	371247
8	12212.6329	439221
9	14163.9736	501635
10	16130.5542	558737
11	18031.9997	608275
12	18292.7491	627214
13		
14		
15		

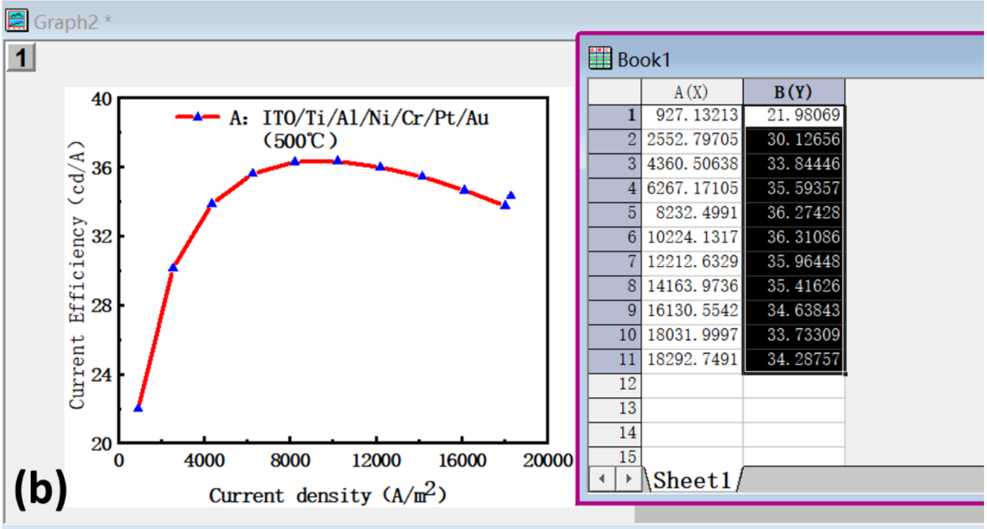

	A(X)	B(Y)
1	927.13213	21.98069
2	2552.79705	30.12656
3	4360.50638	33.84446
4	6267.17105	35.59357
5	8232.4991	36.27428
6	10224.1317	36.31086
7	12212.6329	35.96448
8	14163.9736	35.41626
9	16130.5542	34.63843
10	18031.9997	33.73309
11	18292.7491	34.28757
12		
13		
14		
15		

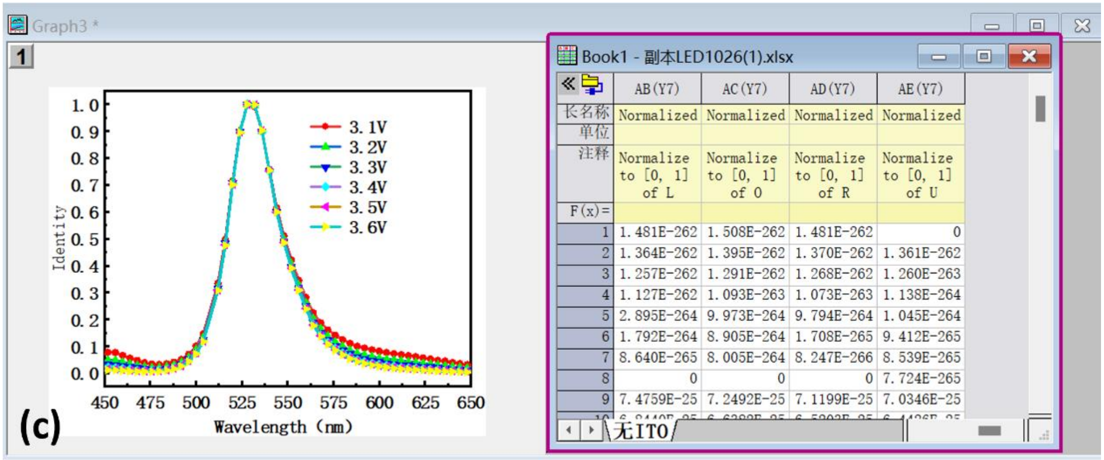

Figure 3s. The optical properties of the device. **(a)** the light brightness-voltage graph of device A. **(b)** the efficiency-current density graph of device A. **(c)** The normalized emission wavelength-voltage graph of the device A
